# Spectral Photon-Counting Computed Tomography (SPCCT): in-vivo single-acquisition multi-phase liver imaging with a dual contrast agent protocol

**DOI:** 10.1038/s41598-019-44821-z

**Published:** 2019-06-11

**Authors:** Salim Si-Mohamed, Valérie Tatard-Leitman, Alexis Laugerette, Monica Sigovan, Daniela Pfeiffer, Ernst J. Rummeny, Philippe Coulon, Yoad Yagil, Philippe Douek, Loic Boussel, Peter B. Noël

**Affiliations:** 1 Univ Lyon, INSA‐Lyon, Université Claude Bernard Lyon 1, UJM-Saint Etienne, CNRS, Inserm, CREATIS UMR 5220, U1206, F‐69621 Lyon, France; 2 Radiology Department, Hospices Civils de Lyon, Lyon, France; 3 Department of Diagnostic and Interventional Radiology, School of Medicine, Technical University of Munich, Munich, Germany; 4 CT Clinical Science, Philips, Suresnes France; 5 Global Advanced Technologies, CT, Philips, Haifa Israel; 6 Department of Radiology, Perelman School of Medicine, University of Pennsylvania, Philadelphia, USA

**Keywords:** Computed tomography, Preclinical research

## Abstract

Diagnostic imaging of hepatocellular carcinoma (HCC) requires a liver CT or MRI multiphase acquisition protocol. Patients would benefit from a high-resolution imaging method capable of performing multi-phase imaging in a single acquisition without an increase in radiation dose. Spectral Photon-Counting Computed Tomography (SPCCT) has recently emerged as a novel and promising imaging modality in the field of diagnostic radiology. SPCCT is able to distinguish between two contrast agents referred to as multicolor imaging because, when measuring in three or more energy regimes, it can detect and quantify elements with a K-edge in the diagnostic energy range. Based on this capability, we tested the feasibility of a dual-contrast multi-phase liver imaging protocol via the use of iodinated and gadolinated contrast agents on four healthy New Zealand White (NZW) rabbits. To perform a dual-contrast protocol, we injected the agents at different times so that the first contrast agent visualized the portal phase and the second the arterial phase, both of which are mandatory for liver lesion characterization. We demonstrated a sensitive discrimination and quantification of gadolinium within the arteries and iodine within the liver parenchyma. In the hepatic artery, the concentration of gadolinium was much higher than iodine (8.5 ± 3.9 mg/mL *versus* 0.7 ± 0.1 mg/mL) contrary to the concentrations found in the liver parenchyma (0.5 ± 0.3 mg/mL *versus* 4.2 ± 0.3 mg/mL). In conclusion, our results confirm that SPCCT allows *in-vivo* dual contrast qualitative and quantitative multi-phase liver imaging in a single acquisition.

## Introduction

Hepatocellular carcinoma (HCC) is the most common primary malignant lesion of the liver and a leading neoplasic death-related cause worldwide^1^. A sensitive imaging modality is required to prevent delay in diagnosis and alter the prognosis of individual patients. Computed tomography (CT) and magnetic resonance imaging (MRI) are the methods of choice in the clinical arena. Diagnostic imaging relies on specific imaging features including amongst others the distinct hepatic circulatory phases, i.e. the arterial and portal phases^2^. However, CT multiphasic imaging is associated with an increase in radiation dose compared to single-phase acquisitions. Multiphasic MRI is associated with high cost, low availability and low spatial resolution. In addition, a multiphasic acquisition can suffer from mis-alignment between the hepatic circulatory phases. This can lead to a lower rate of assessment for especially smaller lesions, explaining why the detection rate of HCC, particularly for lesions <2 cm in diameter, is poor^3–5^. Therefore, HCC diagnostics would benefit from a high-resolution imaging method capable of performing a multi-phase protocol within a single acquisition without increasing the dose of radiation dose or appearance of misalignment artifacts.

Spectral Photon-Counting Computed Tomography (SPCCT) is emerging as a novel and promising imaging modality in the field of diagnostic radiology. Its energy resolving sensors, known as photon-counting detectors (PCDs), enable the analysis of each photon by dividing them into multiple energy bins^6–11^. It is therefore possible to perform not only a two-material decomposition (e.g. water and iodine) but also K-edge imaging to generate an additional material specific map^6,10,12–15^. K-edge imaging is essential for distinguishing between two contrast agents. This characterization enables the obtaining of specific information on elements like gadolinium (E_k_ = 50.2 keV) that have a K-edge in the energy range of CT imaging (≈40–120 keV)^16,17^. Taking advantage of the K-edge imaging approach, some animal studies have recently reported that SPCCT can differentiate between two contrast agents *in-vivo*, e.g. by combining them simultaneously within the vascular and peritoneal compartments^9^ or by injecting them intravenously^18,19^. Another significant benefit of spectral imaging is the possibility to measure the absolute concentration of contrast agents such as gadolinium, iodine, or gold^13,20–22^. Finally, the high spatial resolution of PCDs enables the differentiation of small structures like parenchymal arteries or lesions with sizes below the detection limit of conventional CT^7,23,24^. Accordingly, Mullner *et al*. performed a simulation study demonstrating the feasibility of gold-specific spectral CT imaging for the detection of liver lesions as small as 0.35 × 0.35 cm^2^ in humans^25^.

Altogether, SPCCT seems particularly appropriate for diagnostics of the liver. Past studies were performed using a single-phase acquisition, which, as mentioned above, is not always sufficient for the detection of small liver lesions^3–5^. In a previous study, a hepatic SPCCT in-silico study was performed for simultaneous mapping of two contrast agents, e.g. gadolinium and iodine distribution^26^. In this article, the authors concluded that dual-contrast SPCCT should enable the visualization of the characteristic arterial and portal venous enhancement within a single scan. Although this work employed sophisticated tools and incorporated realistic physical models of an SPCCT system, the hypothesis remains to be confirmed in an *in-vivo* setup. The purpose of this work was to perform an animal study to demonstrate the *in-vivo* feasibility of single-acquisition dual-contrast multi-phase hepatic K-edge imaging with a SPCCT system.

## Materials and Methods

### Spectral photon-counting computed tomography

This study was performed using a modified clinical SPCCT prototype system (Philips, Haifa, Israel) equipped with a photon-counting detector (PCD) which provides up to five consecutive energy windows between 30 and 120 keV^27^. The system uses a conventional x-ray tube which supports three tube voltages, delivers after primary filtering an x-ray spectrum ranging from 30 to 120 keV at 120 kVp, and has tube currents of up to 100 mA. The detection system includes Cadmium Zinc Telluride sensors of two mm thickness and flip-chip bonded to the manufacturer’s proprietary ChromAIX2 ASICs in 28 individual detector tiles^27^. The system supports both axial and helical scan modes with a gantry rotation time of one second with 2400 projection readings per rotation. The field-of-view (FOV) is 168 mm in-plane, with a z-coverage of 2.5 mm in the scanner iso-center. Further technical details concerning the prototype system are provided in a previous study^9^. In the current study, axial scans over 360 degrees were performed at 120 kVp tube voltage, 100 mA tube current and one second gantry rotation time. Energy thresholds were set to 30, 51, 64, 72, 85 keV based on the K-edge of gadolinium at 50.2 keV^28^.

### Animal preparation

This study was approved by the local Institutional Animal Care and Use Committee (CELyne, Council Directive No. 2010/63/UE on the protection of animals used for a scientific purpose) under the authorization n° APAFIS#1732-2015091411181645v3 and performed under relevant guidelines and regulations. Four adult New Zealand White rabbits (Charles River, Canada, mean weight = 3.3 ± 0.4 kg; two females, two males; mean age = 8.4 ± 2.9 months) were sedated before imaging using a 20 mg/kg injection of ketamine (10 mg/ml, Merial, Lyon, France) and 0.25 mg/kg injection of medetomidine (1.0 mg/ml, Orion Pharma, Orion Corporation, Espoo, Finland) in order to maintain general anesthesia for 30 minutes. A bag filled with water was placed on the rabbit to generate homogeneous image noise in the soft-tissue regions.

### Imaging protocol

An 18-gauge catheter was placed in the ear vein of the rabbits for contrast agent administration. Iodine- (1.5 mL/kg, Iomeron, 400 mg/mL, Bracco, Milan, Italy) and gadolinium-based (5 mL/kg, 0.5 M, gadoteridol, Prohance, Bracco, Milan, Italy) contrast agents were injected successively 12 seconds apart with a flow rate at 2 mL/s, starting with the iodine, and the acquisition was performed 9 seconds after the gadolinium injection. Axial scans were acquired at the level of the mid-liver in order to record arterial enhancement. The optimal times of injection and acquisition were determined by preliminary experiments to this study by acquiring multiple acquisitions to determine the peak concentrations of each contrast agent within the different regions of the liver.

SPCCT images and contrast material images, such as iodine or gadolinium maps, were reconstructed with an isotropic voxel size of 250 µm, a matrix size of 640 × 640 pixels and a FOV of 160 mm using a soft reconstruction kernel. FBP reconstructions were used without further post-processing apart from de-ringing, as well as smoothing of the contrast material maps with a Gaussian kernel (width: two pixels). This step decreases the image noise (standard deviation of the mean) by a factor of two to four times. The physical properties of each image pixel are modeled using the concept of projection domain material decomposition and reconstructed from SPCCT data^14^. The concentration of contrast agents at regions-of-interest can then be quantified. Hence, the material decomposition into water, iodine and gadolinium make it possible to quantify the spatial distribution of the contrast agent on a pixel basis. In a more mathematical description, SPCCT therein enables the analysis in a 3-dimensional vector space, the first and second dimensions being the two basis functions independent of the K-edge and the third being provided by K-edge imaging^17,29^. Further details of the reconstruction process can be found in one previous study^10^. Overlay images of the SPCCT and contrast material maps were generated using an image processing software (ImageJ, National Institutes of Health, United States)^30^.

### Contrast agent analysis

**Figure 1:**
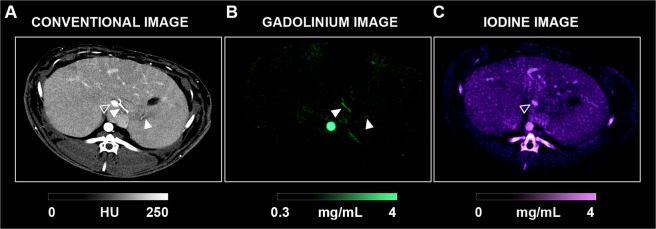
SPCCT dual phase imaging of the liver (**A**: conventional image, **B**: gadolinium image, **C**: iodine image). The hepatic artery (white arrowheads) and the portal vein (empty arrowheads) are enhanced specifically on the gadolinium and iodine maps, respectively, in favor of two enhancement phases. Visual saturation of the enhanced vessels on the conventional images is noticeable due to the selected window/level.

Iodine and gadolinium concentrations in the regions of interest (ROIs) (aorta, proximal hepatic artery, portal vein, subhepatic vein, and liver parenchyma) were obtained by manually drawing circular ROIs of at least 50 pixels by using ImageJ for each rabbit (Fig. 1). Furthermore, for testing the homogeneity of iodine distribution within the liver, three segments of interests were drawn based on the portal vein anatomy, such as it is performed in clinical imaging, including the parenchyma, vessels and biliary system. All ROI’s were defined on conventional images prior to retrieval of the gadolinium and iodine concentrations per organ to avoid operator bias. The ROIs were manually placed by a senior radiologist (SSM, 8 years of experience) in homogeneous regions of the structures of interest and were automatically copied onto the contrast material maps. The data obtained represented the absolute mean concentration of gadolinium and iodine in mg/mL ± standard deviation.

Contrast-to-noise ratios (CNR) and signal-to-noise ratios (SNR) among the four rabbits in each protocol were calculated as follows:

$${\rm{CNR}}=\frac{|{\rm{mean}}\,{\rm{CT}}\,{{\rm{number}}}_{{\rm{hepatic}}{\rm{artery}}}-{\rm{mean}}\,{\rm{CT}}\,{{\rm{number}}}_{{\rm{portal}}{\rm{vein}}}|}{{{\rm{SD}}}_{({\rm{water}})}}$$

$${\rm{SNR}}=\frac{{\rm{mean}}\,{\rm{CT}}\,{{\rm{number}}}_{{\rm{ROI}}}}{{{\rm{SD}}}_{({\rm{water}})}}$$

The standard deviation of the water bag attenuation placed on the rabbit was used as a noise reference.

### Cluster analysis

The simultaneous acquisition of arterial and portal venous phase enables a pixel-by-pixel analysis. Resulting maps from material decomposition (e.g. iodine and gadolinium) may be processed by cluster analysis or support vector machines. In our case, iodine, gadolinium and water can be understood as a three-dimensional (3D) vector space^26^. By plotting the data in 3D space for different ROIs (hepatic artery, portal vein and liver as described above), clusters can be detected which represent a similar tissue type.

### Statistical analysis

To test the homogeneity of the iodine distribution within the liver, a Friedman test was performed.

## Results

Four New Zealand White (NZW) rabbits were scanned after receiving successive injections of iodine and gadolinium-based contrast agents.

Representative conventional, iodine and gadolinium images are shown in Fig. 1. In the conventional images, contrast enhancement generated visualization of both the portal vein and hepatic artery without clear differentiation based on HU values. On the contrary, the signal enhancement on the gadolinium map was specifically localized to the hepatic artery and aorta (arterial phase) while the signal enhancement on the iodine map was specifically localized to the portal vein and liver (portal phase).

**Figure 2:**
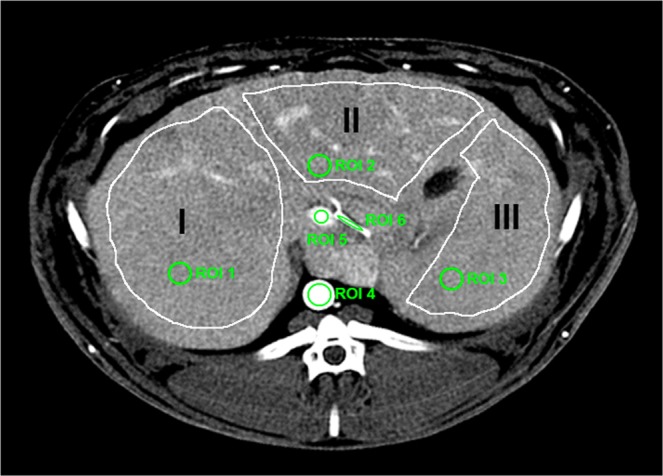
Liver segmentation and ROIs. Three segments of interest (I, II, III) were defined within the liver to test for homogeneity of contrast agent distribution. ROIs were chosen within each liver segment for the parenchyma (ROI 1 to 3), within the aorta (ROI 4), the portal vein (ROI 5) and the hepatic artery (ROI 6) for quantification of iodine and gadolinium.

**Figure 3:**
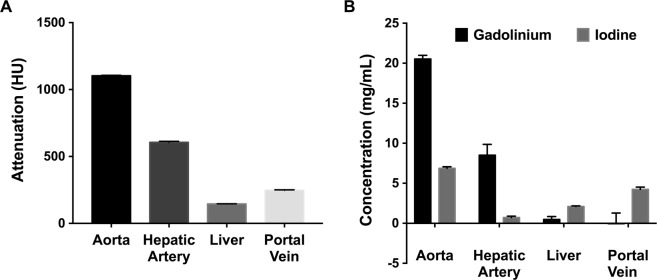
(**A**) Attenuation values (HU) and (**B**) concentrations (mg/mL) of gadolinium and iodine in the aorta, hepatic artery, portal vein and liver.

As shown in Figures 2, 3 and Table 1, the attenuation values and concentrations of gadolinium and iodine were calculated in the liver (ROIs 1 to 3), aorta (ROI 4), portal vein (ROI 5) and hepatic artery (ROI 6). The attenuation values (Fig. 3A) were highest in the aorta (1103.1 ± 108.0 HU), lower in the hepatic artery (605.6 ± 171.9 HU) and portal vein (253.5 ± 49.0 HU), and lowest in the liver parenchyma (149.2 ± 18.25 HU). The concentrations of gadolinium and iodine (Fig. 3B) were highest in the aorta when compared to other regions, with the concentration of gadolinium being approximately three times higher than the concentration of iodine (gadolinium = 20.5 ± 2.07 mg/mL; iodine = 6.9 ± 0.5 mg/mL). Similarly, in the hepatic artery, the concentration of gadolinium was much higher (approximately 10 times) than the concentration of iodine (gadolinium = 8.5 ± 3.9 mg/mL; iodine = 0.7 ± 0.1 mg/mL). On the contrary, in the portal vein and the liver, the concentrations of gadolinium were extremely low compared to the concentrations of iodine as expected from the injection and acquisition timing (portal vein: gadolinium = 0.0 ± 1.3 mg/mL, iodine = 4.2 ± 0.3 mg/mL; liver: gadolinium = 0.5 ± 0.3 mg/mL, iodine = 2.1 ± 0.1 mg/mL).

**Table 1:** Mean attenuations and concentrations of Gadolinium and Iodine in the aorta, hepatic artery, portal vein and liver.

Rabbits N = 4	Attenuation (HU)	Concentration of Gadolinium (mg/mL)	Concentration of Iodine (mg/mL)
Aorta	1103.1 ± 108.0	20.5 ± 2.07	6.9 ± 0.5
Hepatic Artery	605.6 ± 171.9	8.5 ± 3.9	0.7 ± 0.1
Portal Vein	253.5 ± 49.0	0 ± 1.3	4.2 ± 0.3
Liver	149.2 ± 18.25	0.5 ± 0.3	2.1 ± 0.1

Results are expressed as mean ± standard error of the mean of the four rabbits.

As illustrated in Fig. 2, three segments were delineated within the liver, excluding the area of the aorta and the hepatic artery, and the attenuation (HU) in conventional images as well as the concentration of iodine were calculated (Table 2). Statistical analysis showed no differences of attenuation and concentration values between the three liver segments (attenuation: p = 0.93, iodine concentration: p = 0.52), suggesting a homogeneous signal within the liver parenchyma.

**Table 2:** Enhancement within the liver segments of interests.

Segments	Attenuation (HU)			Concentration of Iodine (mg/mL)		
	I	II	III	I	II	III
Rabbit 1	174.1 ± 42.3	154.5 ± 52.5	180.1 ± 49.1	2.4 ± 1.2	2.2 ± 1.6	3.2 ± 1.4
Rabbit 2	134.6 ± 40.5	130.6 ± 47.3	130.3 ± 40.4	1.8 ± 1.5	1.6 ± 1.8	1.7 ± 1.5
Rabbit 3	135.8 ± 38.5	121.6 ± 44.5	121.5 ± 39.9	1.8 ± 0.7	1.5 ± 0.7	1.7 ± 0.6
Rabbit 4	161.2 ± 44.5	173.4 ± 42.0	186.5 ± 47.4	2.4 ± 0.9	2.5 ± 1.1	2.4 ± 1.0

The results are expressed as mean ± the standard deviation.

**Figure 4:**
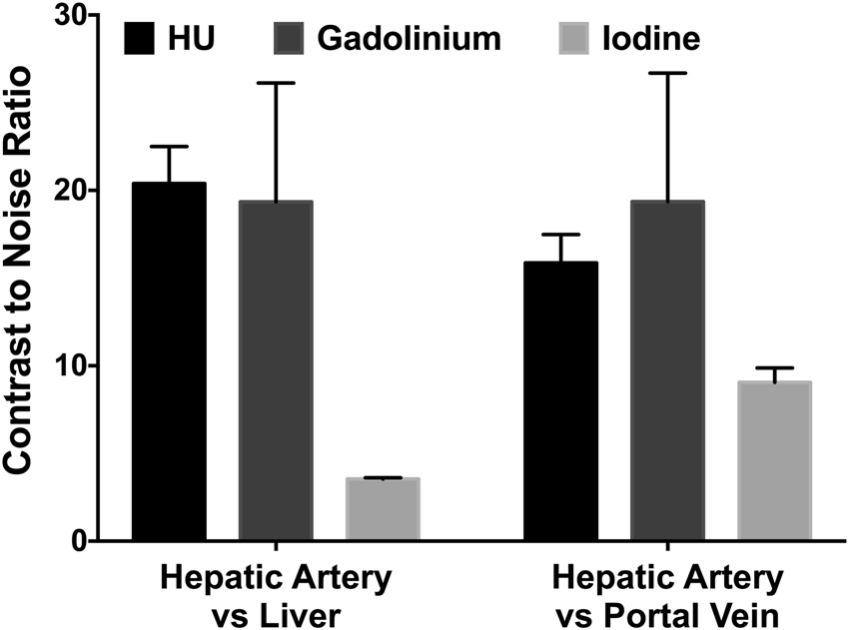
Contrast-to-Noise Ratio for hepatic artery versus liver and hepatic artery versus portal vein in conventional (HU) images as well as gadolinium and iodine maps. Despite the suitable CNR on the HU images, only the spectral images allow a specific visualization of each phase of hepatic enhancement.

The CNR were calculated for the regions of interest: hepatic artery versus liver and hepatic artery versus portal vein for the conventional images as well as the gadolinium and iodine maps, as shown in Fig. 4. The CNR for hepatic artery versus liver and hepatic artery versus portal vein were very similar between the conventional images and the gadolinium map while they were approximately six times and two times lower for the iodine map, respectively (CNR hepatic artery versus liver: conventional images = 20.3 ± 2.1, Gadolinium map = 19.3 ± 6.7, Iodine map = 3.5 ± 0.1; CNR hepatic artery versus portal vein: conventional images = 15.8 ± 1.6, gadolinium map = 19.3 ± 7.3, iodine map = 9.0 ± 0.2). Note, while the CNR were similar between the hepatic artery versus liver and hepatic artery versus portal vein for the conventional images and the gadolinium map, it was approximately 2.5 times higher for the hepatic artery versus portal vein compared to hepatic artery versus liver in the iodine map. This is explained by the higher concentration of iodine measured in the portal vein than in the liver as expected during the portal phase.

**Figure 5:**
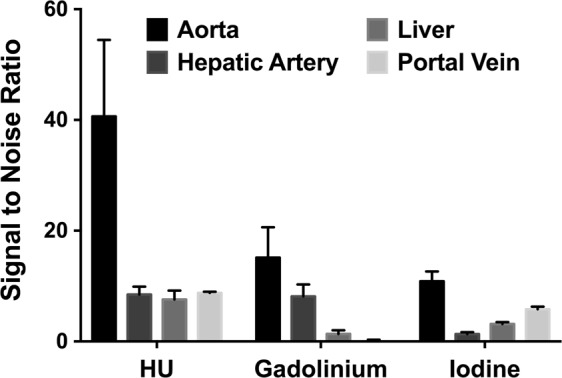
Signal-to-Noise Ratio for the hepatic artery, portal vein and liver in conventional (HU) images and the gadolinium as well as iodine maps.

The SNR for the hepatic artery, portal vein and liver in the conventional images as well as the gadolinium and iodine maps are presented in Fig. 5. The SNR for the hepatic artery, portal vein and liver were very similar in the conventional images (SNR conventional images: hepatic artery = 8.5 ± 1.4; portal vein = 8.8 ± 0.2; liver = 7.6 ± 1.6). On the contrary, in the gadolinium map the SNR was high for the hepatic artery, while it was negligible for the portal vein and the liver (SNR gadolinium map: hepatic artery = 8.1 ± 2.1; portal vein = −0.1 ± 0.4; liver = 1.3 ± 0.6). On the contrary, the SNR for the hepatic artery was lower than the SNR for the portal vein and liver in the iodine map (SNR iodine map: hepatic artery = 1.3 ± 0.3; portal vein = 5.8 ± 0.4; liver = 3.1 ± 0.3).

**Figure 6:**
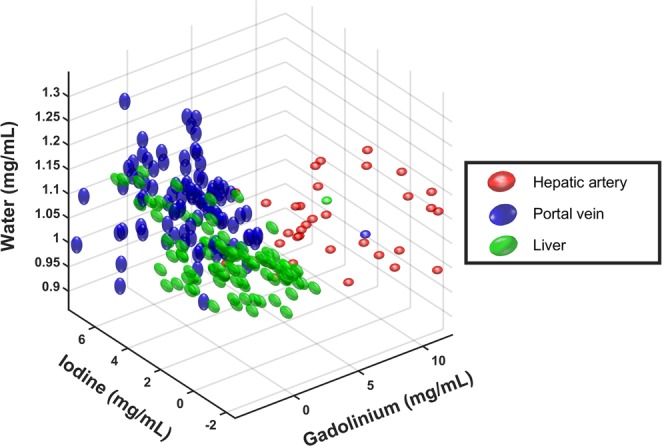
Scatter plot derived from an *in-vivo* acquisition and the sequential decomposition into material maps. Iodine, gadolinium and water can be understood as a three-dimensional (3D) vector space. Hepatic artery (red), portal vein (blue), and liver (green) are showing individual clusters with minimal overlap. Each data point represents a single pixel within the regions of interests.

Figure 6 illustrates an exemplary cluster visualization in the 3D vector space consisting of iodine, gadolinium, and water. The scatter plot derived from an *in-vivo* acquisition shows three separate clusters which can be identified as hepatic artery, portal vein, and liver. As expected, the liver and portal vein clusters are slightly overlapped due to the portal enhancement of the liver. The noticeable difference is the range of iodine concentrations in these two structures.

## Discussion

The present study demonstrates that SPCCT technology can be employed successfully to perform *in-vivo* dual contrast multi-phase imaging in a single acquisition. By injecting iodine and gadolinium-based contrast agents sequentially, a single SPCCT acquisition enabled visualization of the portal venous and arterial phases of the liver of healthy rabbits. To the best of our knowledge, while others and we have already reported *in-vivo* dual contrast agent SPCCT imaging^9,10,18,19^, this study is the first of its kind to report *in-vivo* dual phase hepatic SPCCT imaging.

Current SPCCT technology, by way of multiple energy bins, produces a calculation of material maps based on individual K-edges. In order for spectral CT systems to visualize the K-edge, there must be more than two energy bins^7,10,31^. It is impossible for conventional hepatic CT to capture the portal venous and arterial phases in a way that differentiates both phases on a single image. Utilizing the novel technology of SPCCT, we found that iodine and gadolinium maps showed an enhancement that was mostly restricted to the portal vein and hepatic artery, respectively, enabling clear differentiation. These results were further confirmed by quantifying the contrast agents in the hepatic artery, portal vein and liver. We found that the iodine concentration was high in the portal vein while being very low in the hepatic artery, and that the opposite was apparent when measuring in the gadolinium maps. As expected, we observed a high concentration of iodine in the liver as the portal vein is responsible for two-thirds of the perfusion^32^. Gadolinium enhancement was minimal. This result was foreseeable because the hepatic artery provides one-fifth to one-third of the total hepatic blood flow. In addition, the knowledge of this physiological aspect explains our choice in order of injection for iodine and gadolinium. As the volume of distribution of the arterial compartments is lower than the portal compartments, we chose gadolinium for hepatic artery visualization in order to reduce the dose of gadolinium compared to what would be necessary for imaging the portal compartment. In addition, as part of our preliminary research, we have determined a feasible solution to ensure accurate injection timing^33^. In particular, by performing a bolus tracking procedure with respect to the first contrast agent, the single image acquisition can be optimally timed for the expected maximal contrast uptake of both contrast agents. Starting with a standard dose of iodine allows us to optimize and reduce the dose of gadolinium as a second contrast agent.

A recent study reported in an *in-vitro* experiment that a mixer of gadolinium and iodine could lead to an underestimation in quantification of the K-edge contrast agent (gadolinium)^10^. Advanced iterative reconstruction methods, such as one-step algorithms, may reduce this bias and improve the CNR in liver imaging^34–37^. In our study, in addition to a good SNR for the hepatic artery and portal vein, the CNR values for the hepatic artery versus liver or portal vein with both gadolinium and iodine suggested a satisfactory specificity of the spectral discrimination. This can be explained by the fact that the maps generated via material decomposition mainly represent the enhanced structures without other anatomical information. These results were confirmed by an advanced scatter analysis, which was enabled by a pixel-based spatial alignment. Clusters in the 3D space represent the same tissue type, as one could observe in our results for the hepatic artery, portal vein, and healthy liver tissue.

Our work is the first step in validation of a recent study by Muenzel *et al*., who demonstrated the possibility of performing simultaneous dual-contrast multi-phase liver imaging using SPCCT with an in-silico model^26^. In this earlier study, results were obtained by simulating four types of liver lesions with a characteristic arterial and portal venous pattern (haemangioma, hepatocellular carcinoma, cyst, and metastasis). Results from both studies therefore suggest that SPCCT simultaneous dual contrast multi-phase imaging of liver diseases is feasible for applications in *in-vivo* studies. Liver examinations for diagnosis and follow-up would then require only one acquisition instead of two as is the case today with conventional or dual energy CT. This has two major advantages. First, it would lead to an overall decrease of ionizing radiation delivered to the patient, which is particularly important as the follow up of chronic liver diseases requires recurrent CT scans. Second, as the information of arterial and portal venous phases are derived from a single SPCCT acquisition, it would lead to elimination of post-processing techniques i.e. motion-correction that are necessary for repetitive CT, thereby leading to better assessment of smaller lesions (<2 mm in diameter). This would be a major improvement especially for the identification of small structures such as focal liver lesions (e.g. hepatocellular carcinoma (HCC)). Therefore, SPCCT should allow an early detection of HCC, which unlike the liver parenchyma are mostly perfused by the hepatic artery. Finally, SPCCT will also lead to the possibility to obtain virtually unenhanced images together with the gadolinium and iodine maps, which is important for e.g. visualization of calcifications within the liver.

Our study has limitations. First, there were no lesions to be detected or classified and the number of animals was small. An appropriate experimental approach would be to employ a VX2 tumor model, which is well established for New Zealand White rabbits. Second, spectral analysis, in general, amplifies noise, which is also one of the challenges of dual energy CT^8^. Nevertheless, photon counting has the potential of superior spectral separation compared with dual energy CT, and is therefore expected to enable superior spectral results at same dose levels or similar spectral performance at lower dose levels^7^.

Overall, our work demonstrates that SPCCT could be a promising tool for the characterization and follow up of human liver diseases and treatments. Several parameters have already been evaluated in this study for a successful transition into the clinical routine. As one example, the energy thresholds (30, 51, 64, 72, 85 keV) of the photon-counting detector have already been optimized and tested for the K-edge of gadolinium (50.2 keV)^28^. To determine the best clinical protocol, many other parameters such as the concentrations of contrast agents, their order and timing of injection, the timing of the single acquisition, and the field-of-view of the system will need to be evaluated and determined by future studies.

## Conclusion

Our study validates the feasibility of a multi-phase liver imaging with a dual-contrast agent protocol *in-vivo* using SPCCT to obtain a reasonable assessment of the arterial and portal venous phases of a rabbit’s liver within a single acquisition.

## References

[1] Balogh J (2016). Hepatocellular carcinoma: a review. J. Hepatocell. Carcinoma.

[2] Lurie Y, Webb M, Cytter-Kuint R, Shteingart S, Lederkremer GZ (2015). Non-invasive diagnosis of liver fibrosis and cirrhosis. World J. Gastroenterol..

[3] Forner A (2008). Diagnosis of hepatic nodules 20 mm or smaller in cirrhosis: Prospective validation of the noninvasive diagnostic criteria for hepatocellular carcinoma. Hepatol. Baltim. Md.

[4] Lauenstein TC (2007). Gadolinium-enhanced MRI for tumor surveillance before liver transplantation: center-based experience. AJR Am. J. Roentgenol..

[5] Luca A (2010). Multidetector-row computed tomography (MDCT) for the diagnosis of hepatocellular carcinoma in cirrhotic candidates for liver transplantation: prevalence of radiological vascular patterns and histological correlation with liver explants. Eur. Radiol..

[6] Si-Mohamed S (2017). Review of an initial experience with an experimental spectral photon-counting computed tomography system. Nucl. Instrum. Methods Phys. Res..

[7] Taguchi K, Iwanczyk JS (2013). Vision 20/20: Single photon counting x-ray detectors in medical imaging. Med. Phys..

[8] McCollough CH, Leng S, Yu L, Fletcher JG (2015). Dual- and multi-energy CT: principles, technical approaches, and clinical applications. Radiology.

[9] Si-Mohamed, S. *et al*. Improved Peritoneal Cavity and Abdominal Organ Imaging Using a Biphasic Contrast Agent Protocol and Spectral Photon Counting Computed Tomography K-Edge Imaging. *Invest*. *Radiol*. (2018). 10.1097/RLI.0000000000000483 PMC6291259 29794948

[10] Si-Mohamed S (2018). Multicolour imaging with spectral photon-counting CT: a phantom study. Eur. Radiol. Exp..

[11] Riederer I (2019). Liquid Embolic Agents in Spectral X-Ray Photon-Counting Computed Tomography using Tantalum K-Edge Imaging. Sci. Rep..

[12] Pourmorteza A (2016). Abdominal imaging with contrast-enhanced photon-counting CT: first human experience. Radiology.

[13] de Vries A (2015). Quantitative spectral K-edge imaging in preclinical photon-counting x-ray computed tomography. Invest. Radiol..

[14] Schlomka JP (2008). Experimental feasibility of multi-energy photon-counting K-edge imaging in pre-clinical computed tomography. Phys. Med. Biol..

[15] Riederer I (2019). Differentiation between blood and iodine in a bovine brain-Initial experience with Spectral Photon-Counting Computed Tomography (SPCCT). PloS One.

[16] Roessl E (2011). Sensitivity of photon-counting based K-edge imaging in X-ray computed tomography. IEEE Trans. Med. Imaging.

[17] Roessl E, Proksa R (2007). K-edge imaging in x-ray computed tomography using multi-bin photon counting detectors. Phys. Med. Biol..

[18] Symons R (2017). Photon-counting CT for simultaneous imaging of multiple contrast agents in the abdomen: an *in vivo* study. Med. Phys..

[19] Cormode DP (2017). Multicolor spectral photon-counting computed tomography: *in vivo* dual contrast imaging with a high count rate scanner. Sci. Rep..

[20] Dangelmaier Julia, Bar-Ness Daniel, Daerr Heiner, Muenzel Daniela, Si-Mohamed Salim, Ehn Sebastian, Fingerle Alexander A., Kimm Melanie A., Kopp Felix K., Boussel Loic, Roessl Ewald, Pfeiffer Franz, Rummeny Ernst J., Proksa Roland, Douek Philippe, Noël Peter B. (2018). Experimental feasibility of spectral photon-counting computed tomography with two contrast agents for the detection of endoleaks following endovascular aortic repair. European Radiology.

[21] Si-Mohamed S (2017). Evaluation of spectral photon counting computed tomography K-edge imaging for determination of gold nanoparticle biodistribution *in vivo*. Nanoscale.

[22] Muenzel Daniela, Bar-Ness Daniel, Roessl Ewald, Blevis Ira, Bartels Matthias, Fingerle Alexander A., Ruschke Stefan, Coulon Philippe, Daerr Heiner, Kopp Felix K., Brendel Bernhard, Thran Axel, Rokni Michal, Herzen Julia, Boussel Loic, Pfeiffer Franz, Proksa Roland, Rummeny Ernst J., Douek Philippe, Noël Peter B. (2017). Spectral Photon-counting CT: Initial Experience with Dual–Contrast Agent K-Edge Colonography. Radiology.

[23] Mannil Manoj, Hickethier Tilman, von Spiczak Jochen, Baer Matthias, Henning André, Hertel Madeleine, Schmidt Bernhard, Flohr Thomas, Maintz David, Alkadhi Hatem (2018). Photon-Counting CT. Investigative Radiology.

[24] Kopp FK (2018). Evaluation of a preclinical photon-counting CT prototype for pulmonary imaging. Sci. Rep..

[25] Müllner M, Schlattl H, Hoeschen C, Dietrich O (2015). Feasibility of spectral CT imaging for the detection of liver lesions with gold-based contrast agents - A simulation study. Phys. Med..

[26] Muenzel D (2017). Simultaneous dual-contrast multi-phase liver imaging using spectral photon-counting computed tomography: a proof-of-concept study. Eur. Radiol. Exp..

[27] Steadman R, Herrmann C, Livne A (2017). ChromAIX2: A large area, high count-rate energy-resolving photon counting ASIC for a spectral CT prototype. Nucl. Instrum. Methods Phys. Res. Sect. Accel. Spectrometers Detect. Assoc. Equip..

[28] Roessl E, Herrmann C (2009). Cramér–Rao lower bound of basis image noise in multiple-energy x-ray imaging. Physics in Medicine and Biology.

[29] Roessl, E. E. Imaging performance of a photon-counting computed tomography prototype (CERN, Geneva, Switzerland, 2015).

[30] Schneider CA, Rasband WS, Eliceiri KW (2012). NIH Image to ImageJ: 25 years of image analysis. Nat. Methods.

[31] Willemink MJ, Persson M, Pourmorteza A, Pelc NJ, Fleischmann D (2018). Photon-counting CT: Technical Principles and Clinical Prospects. Radiology.

[32] Eipel C, Abshagen K, Vollmar B (2010). Regulation of hepatic blood flow: The hepatic arterial buffer response revisited. World J. Gastroenterol. WJG.

[33] Proksa, R. *et al*. System for providing images for endoleak detection. Patent WO/2018/00 (2018).

[34] Mechlem K (2018). Joint Statistical Iterative Material Image Reconstruction for Spectral Computed Tomography Using a Semi-Empirical Forward Model. IEEE Trans. Med. Imaging.

[35] Zhang Y (2016). Spectral CT Reconstruction with Image Sparsity and Spectral Mean. IEEE Trans. Comput. Imaging.

[36] Chen B, Zhang Z, Sidky EY, Xia D, Pan X (2017). Image reconstruction and scan configurations enabled by optimization-based algorithms in multispectral CT. Phys. Med. Biol..

[37] Mory, C., Sixou, B., Si-Mohamed, S., Boussel, L. & Rit, S. Comparison of five one-step reconstruction algorithms for spectral CT. *Phy Med Bio* (2018). 10.1088/1361-6560/aaeaf2 30465541

